# Supplementary material for: Near infrared sensitized exciton upconversion luminescence from inorganic perovskite nanocrystals
Source: Nat Commun. 2025 Jul 2;16:6080. doi: 10.1038/s41467-025-61293-0 (PMC12223018; doi:10.1038/s41467-025-61293-0)
Supplement: Supplementary file 1 — Supplementary Information [file 41467_2025_61293_MOESM1_ESM.pdf]

Supplementary Information for

Near Infrared Sensitized Exciton Upconversion from Inorganic Perovskite Nanocrystals

Yiyan Zhang[1,3], Tianyu Zhao[1,3], Yuming Deng[2], Xinyue Liu[2], Xiaorong Zhang[1,3], Tong Zhu[2*], Hans Ågren[1,3,4], Guanying Chen[1,3*]

[1]MIIT Key Laboratory of Critical Materials Technology for New Energy Conversion and Storage, School of Chemistry and Chemical Engineering, Ministry of Education, Harbin Institute of Technology, 150001 Harbin, People's Republic of China.

[2]Laser Micro/Nano Fabrication Laboratory, School of Mechanical Engineering, Beijing Institute of Technology, Beijing 100081, P. R. China.

[3]Key Laboratory of Micro-systems and Micro-structures, Ministry of Education, Harbin Institute of Technology, 150001 Harbin, People's Republic of China.

[4]Department of Physics and Astronomy, Division of X-ray Photon Science, Uppsala University SE-75121 Uppsala, Sweden

Supplementary Figures

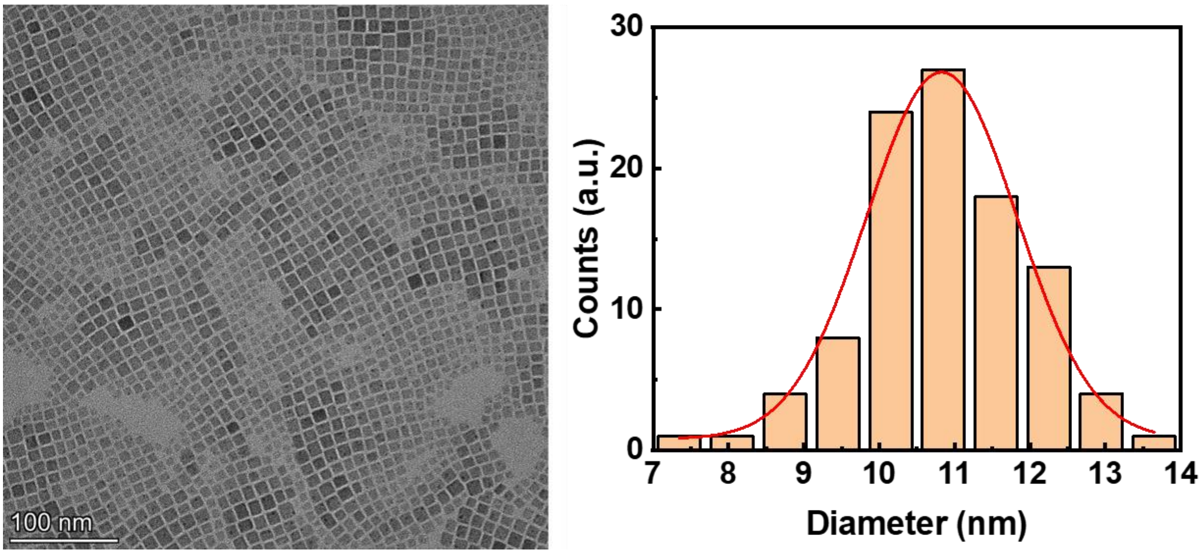

Supplementary Figure 1. Transmission electron microscopy (TEM) image of undoped $CsPbBr_3$ nanocrystals (left) and corresponding size distribution (right).

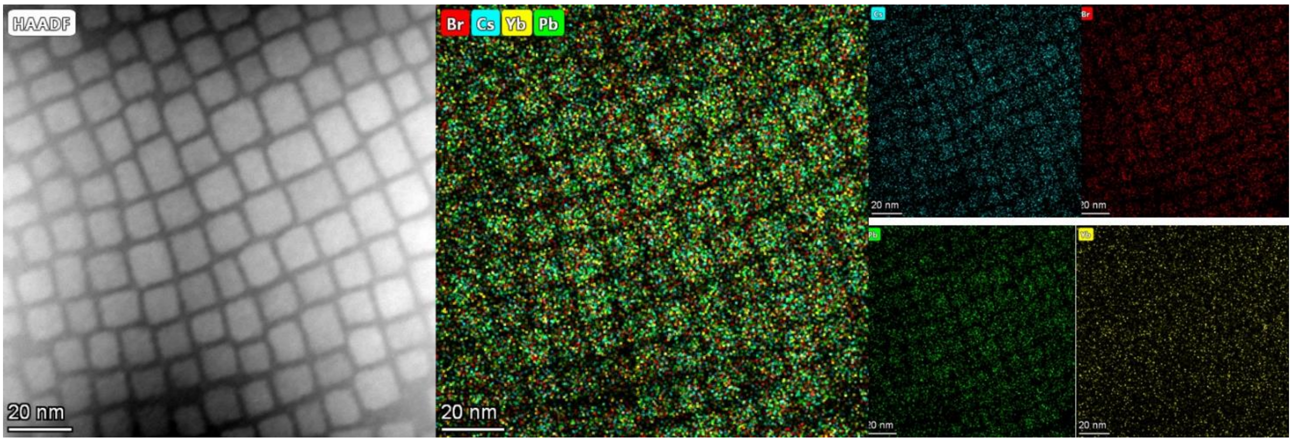

Supplementary Figure 2. High-angle annular dark-field scanning transmission electron microscopy (HADDF-STEM) image (left), overlapped energy dispersive x-ray spectroscopy (EDS) mapping (middle), and corresponding EDS mapping of each involved elements (right) in Yb^{3+}-doped $CsPbBr_3$ nanocrystals.

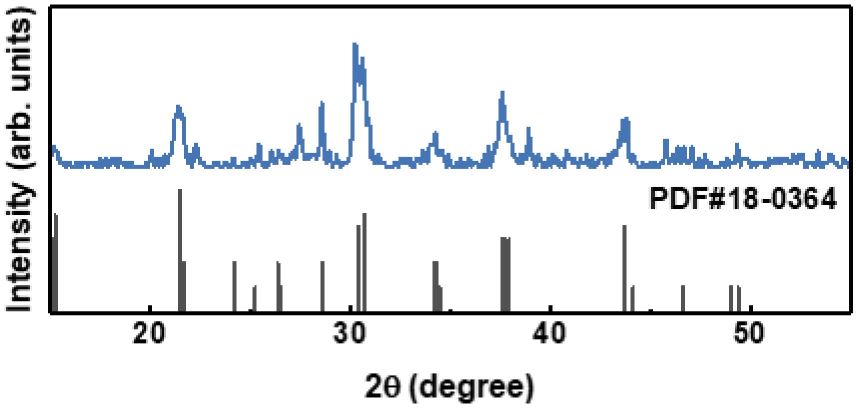

Supplementary Figure 3. X-ray diffraction (XRD) pattern of $CsPbBr_3:Yb^{3+}$ nanocrystals contrasted with the standard diffraction pattern of a monoclinic crystal phase (PDF 18-0364) as a reference.

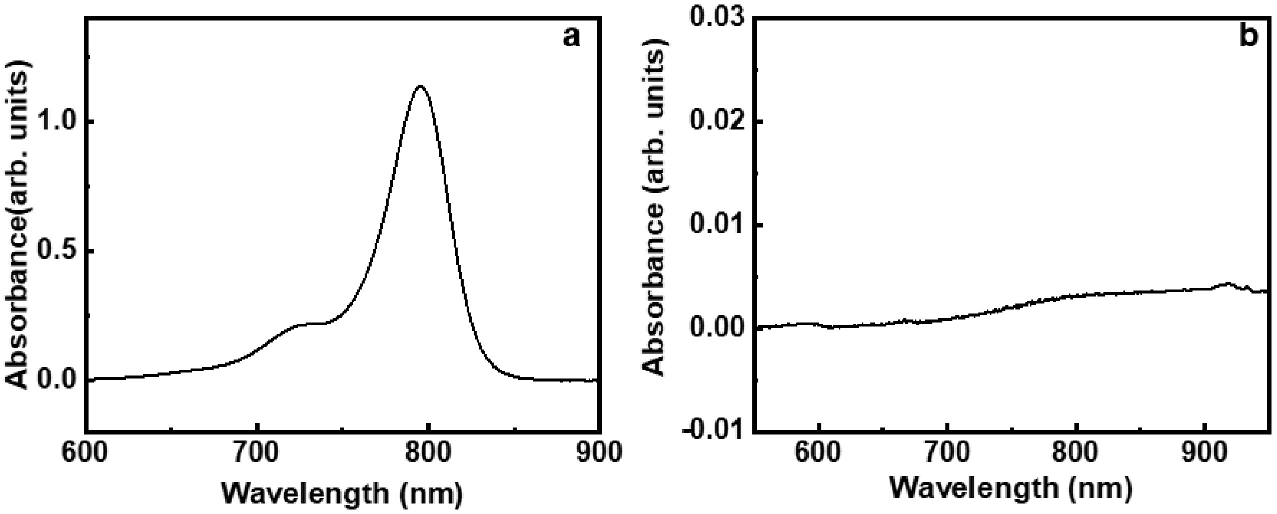

Supplementary Figure 4. Absorbance of IR783 in chloroform (a) and in cyclohexane (b).

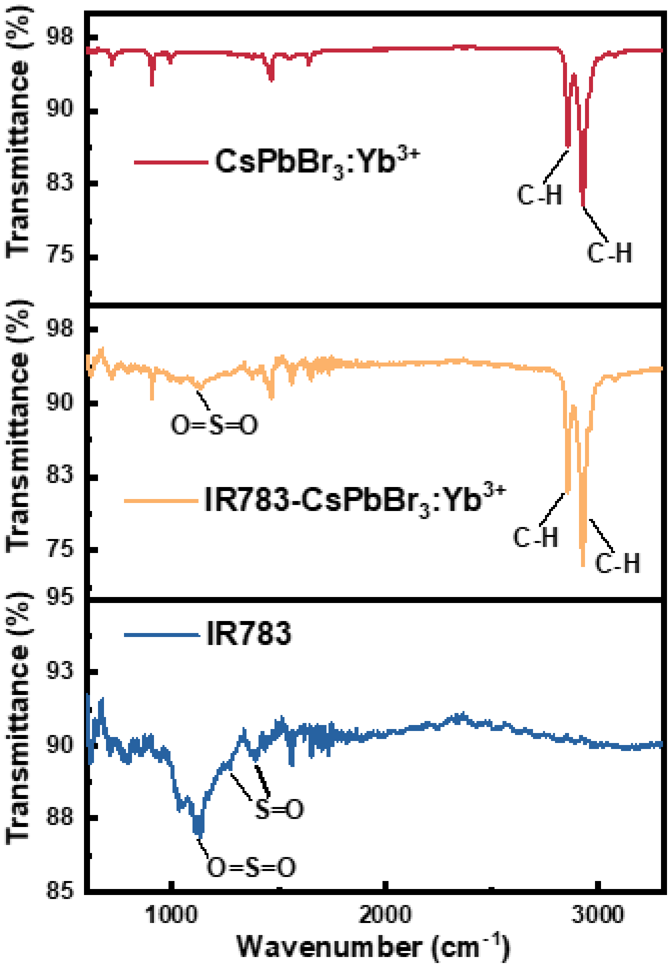

Supplementary Figure 5. Fourier transform infrared spectroscopy of $CsPbBr_3:Yb^{3+}$ (top), IR783-$CsPbBr_3:Yb^{3+}$ (middle) and IR783 (bottom).

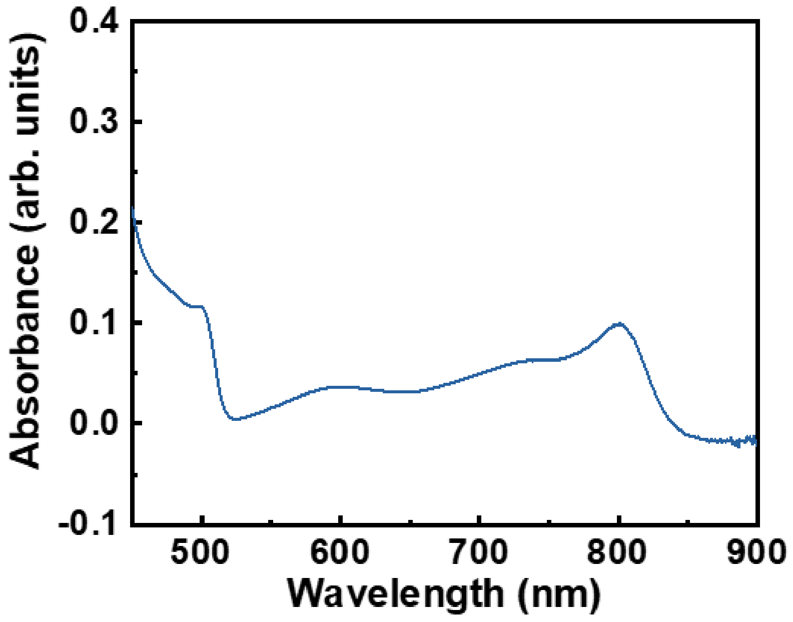

Supplementary Figure 6. Absorption spectrum of $CsPbBr_3$-Yb^{3+}-IR780

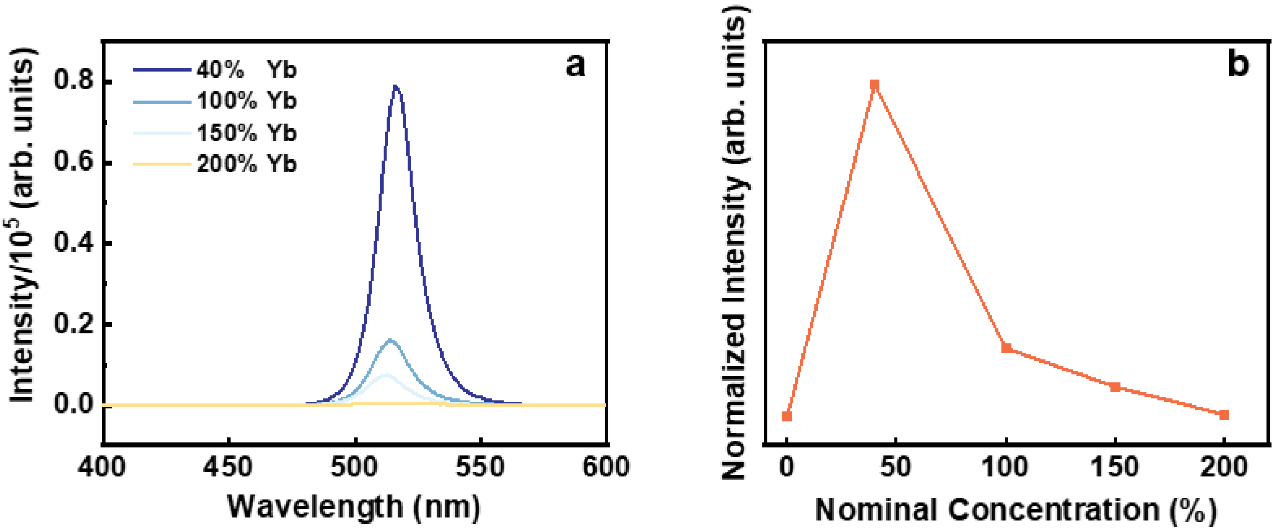

Supplementary Figure 7. Upconversion luminescence spectra of IR783-$CsPbBr_3$:Yb^{3+} nanocrystals with varied doped Yb^{3+} concentrations (a) and the corresponding intensity change at peak wavelength (b). Excited under 804 nm laser, power density= 8.4 W/cm^2.

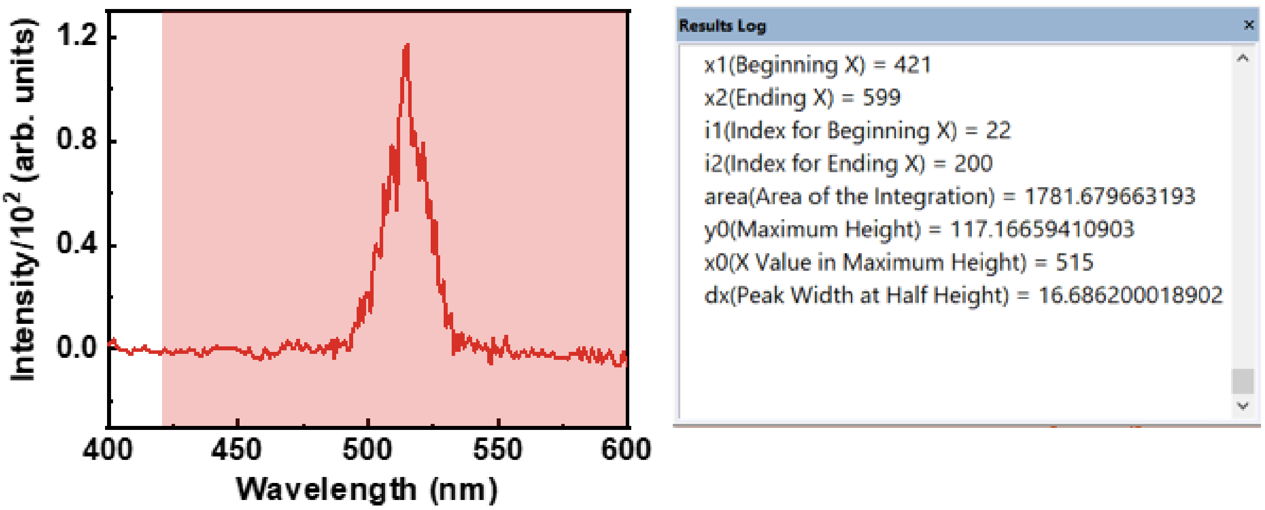

Supplementary Figure 8. Integrated upconversion luminescence intensity of $CsPbBr_3:Yb^{3+}$ nanocrystal (from 400-600 nm) under continuous-wave laser irradiance at 980 nm (8.4 W/cm^2), corresponding to the absorption of Yb^{3+} dopants.

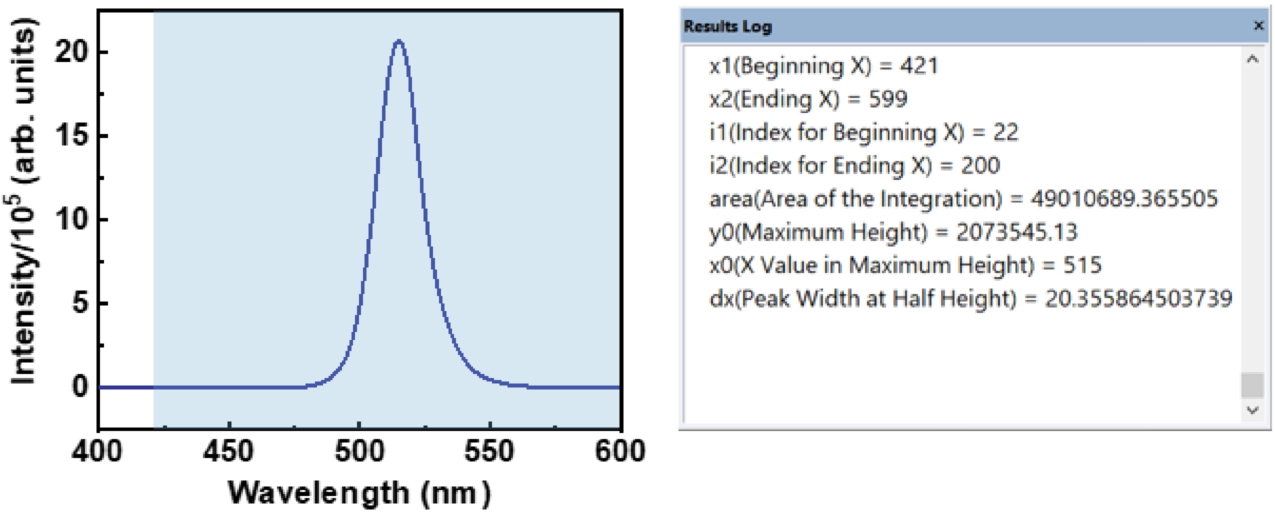

Supplementary Figure 9. Integrated upconversion luminescence intensity of the IR783-$CsPbBr_3:Yb^{3+}$ coupled system (from 400-600 nm) under continuous-wave laser irradiance at 804 nm (8.4 W/cm^2), corresponding to the absorption of IR783 dye.

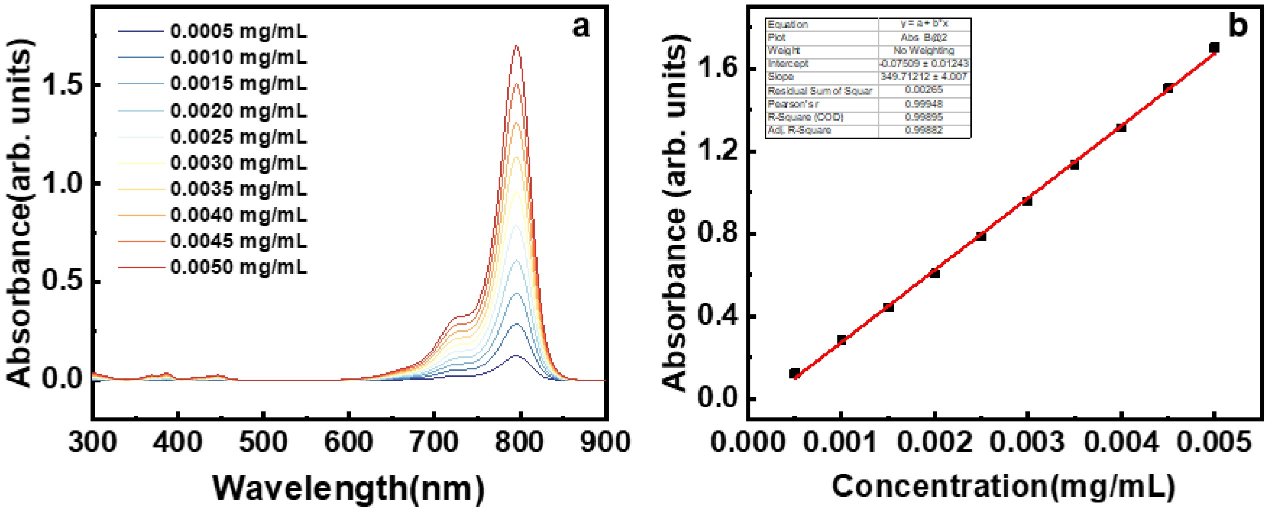

Supplementary Figure 10. Absorbance of IR783 with different concentrations (a); the linear dependence of the absorbance on IR 783 dye concentration (b).

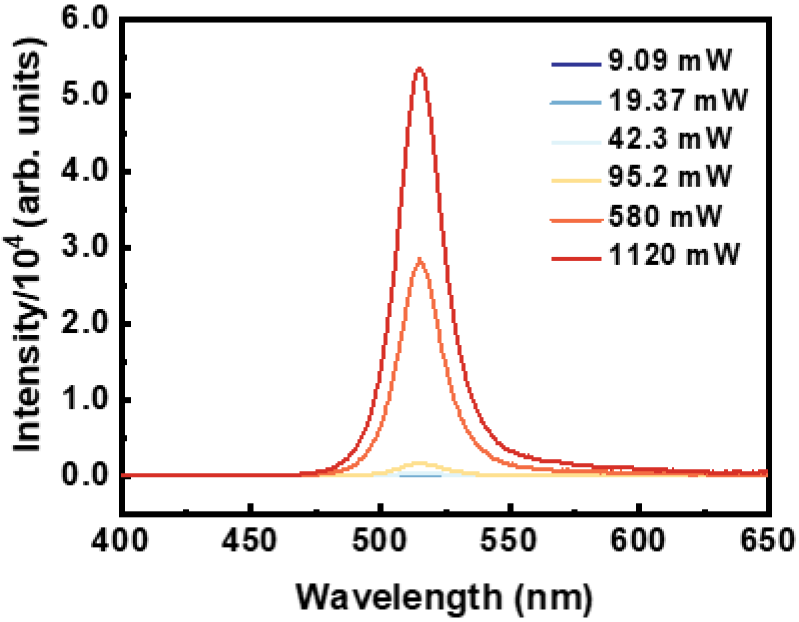

Supplementary Figure 11. Power-dependent upconversion luminescence spectra of IR783-$CsPbBr_3$:Yb^{3+} nanocrystals under continuous-wave 804 nm laser excitation.

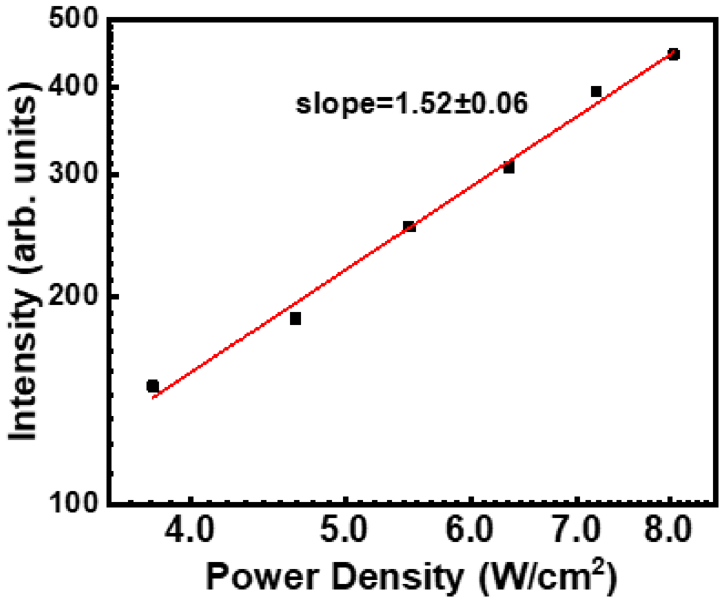

Supplementary Figure 12. The dependence of integrated exciton upconversion luminescence on pump power density of $CsPbBr_3:Yb^{3+}$ nanocrystals in logarithmic scale; excitation at 980 nm. A slope of 1.52 ± 0.06 was obtained by linear fitting (red solid line) of the experimental data (black square), indicating a two-photon process involved to produce the exciton upconversion luminescence.

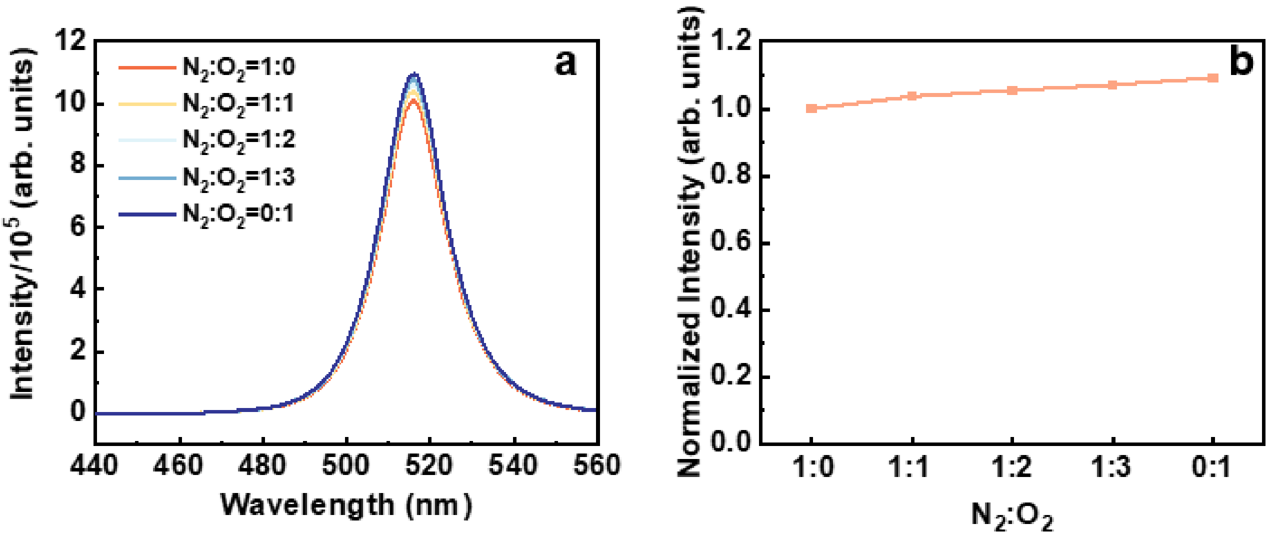

Supplementary Figure 13. Stokes luminescence spectra of $CsPbBr_3$:Yb^{3+} at atmosphere with different ratios of N_2:O_2 (a) and its intensity change at peak wavelength (b). Excitation at 365 nm.

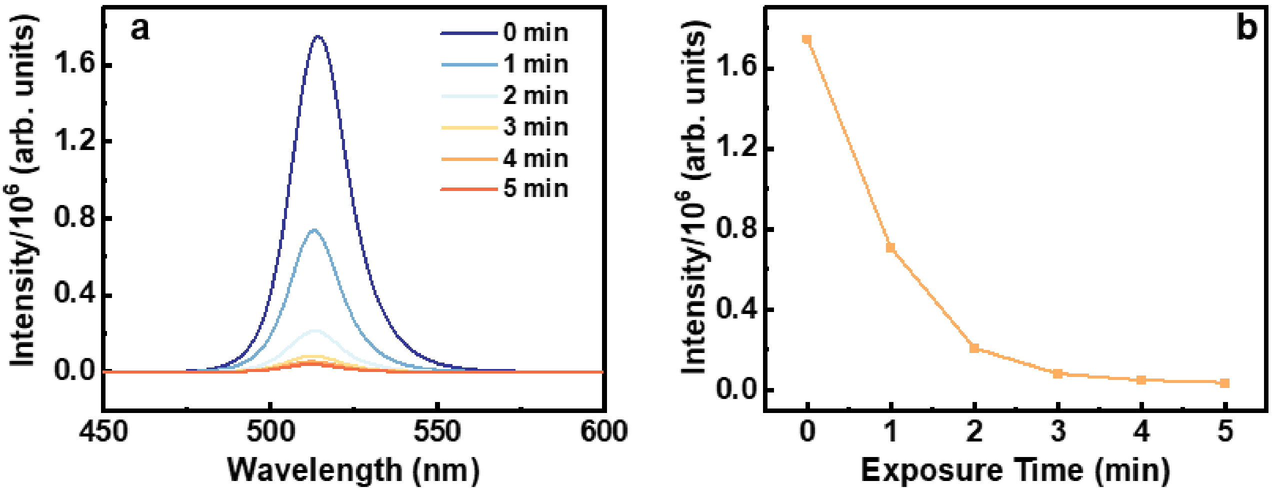

Supplementary Figure 14. Upconversion luminescence spectra (left) of IR783-$CsPbBr_3:Yb^{3+}$nanocrystals following exposure in air at different time (a), with corresponding peak intensity change at 515 nm (b). Excited at 804 nm, power density = 8.4 W/cm^2.

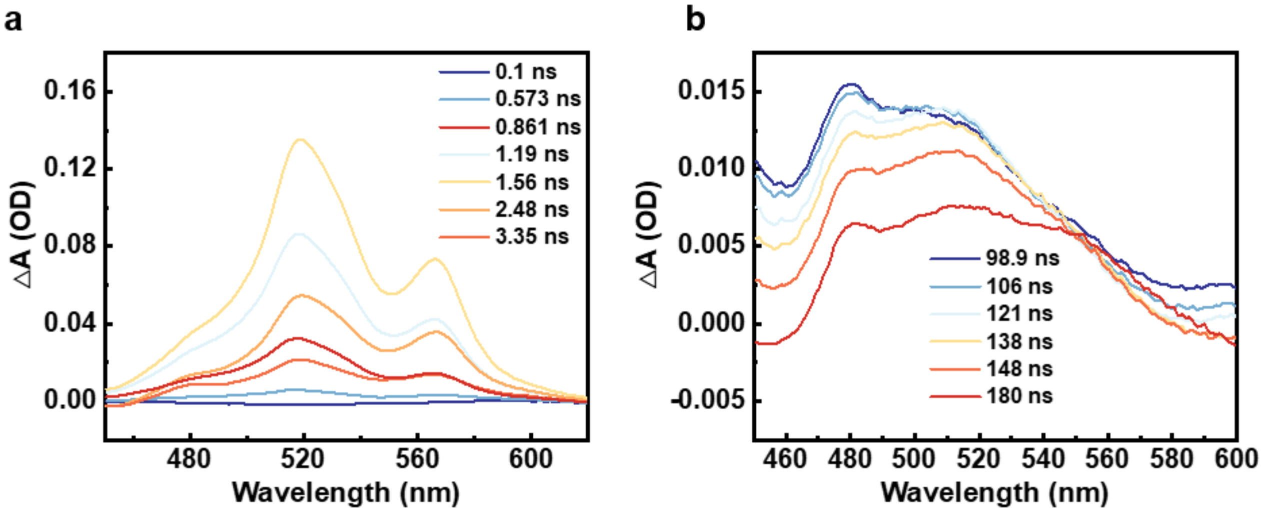

Supplementary Figure 15. Transient absorption spectra of singlet (a) and triplet (b) of IR783 in IR783-$CsPbBr_3$.

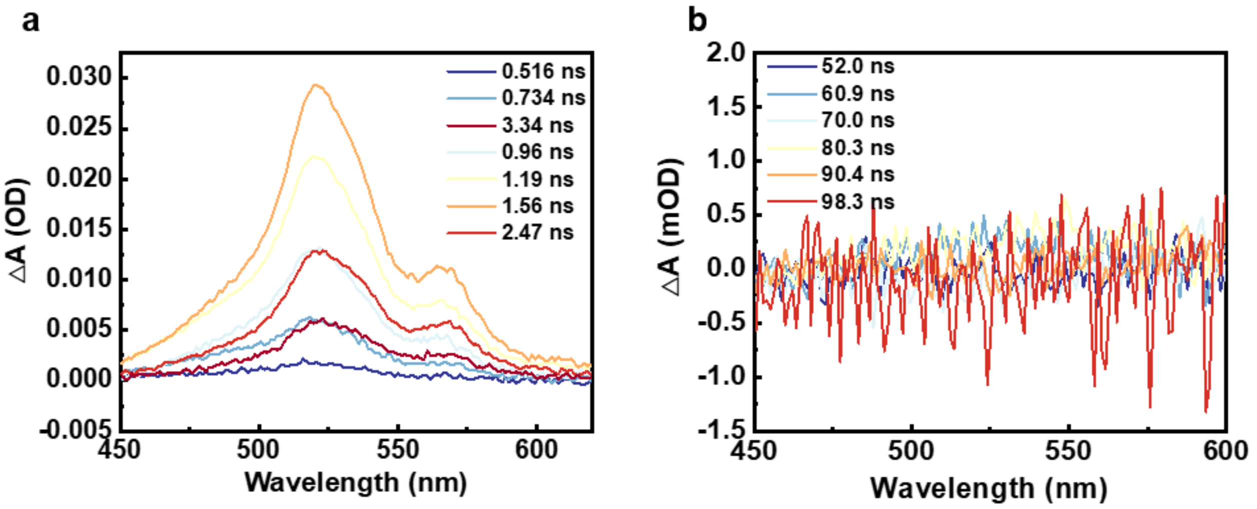

Supplementary Figure 16. Transient absorption spectra of IR783-$CsPbBr_3$:Yb^{3+} at (a) short and (b) longer timescale. Note that panel (b) uses milli-optical density (mOD), in contrast to optical density (OD) in panel (a), to provide an enlarged view of the measured signal.

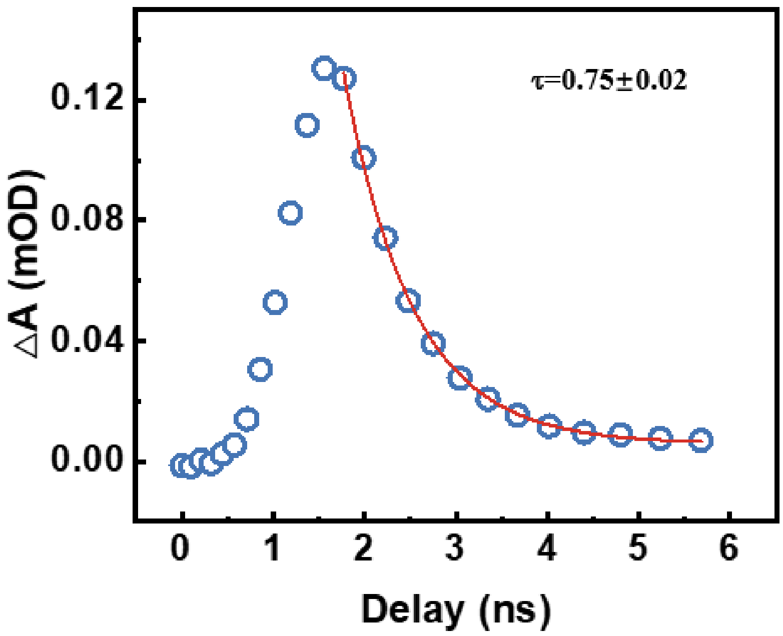

Supplementary Figure 17. Time-resolved singlet excited state absorption process of IR783 dye (522 nm) at the stage of first 5 ns. An estimated singlet lifetime of 0.75±0.02 ns was acquired by fitting the decay process (red solid line).

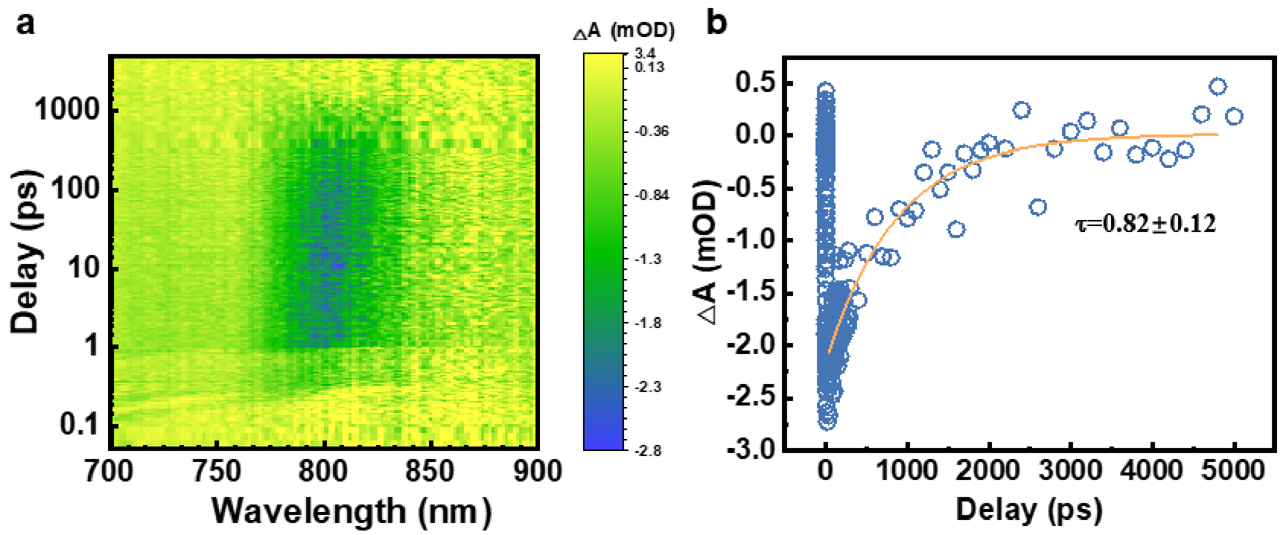

Supplementary Figure 18. Femtosecond transient absorption of IR783-$CsPbBr_3$ nanocrystals (a), and measured decay process of the ground state bleaching at 800 nm (b). An estimated singlet lifetime of 0.82±0.12 ns was acquired by fitting the decay process (red solid line).

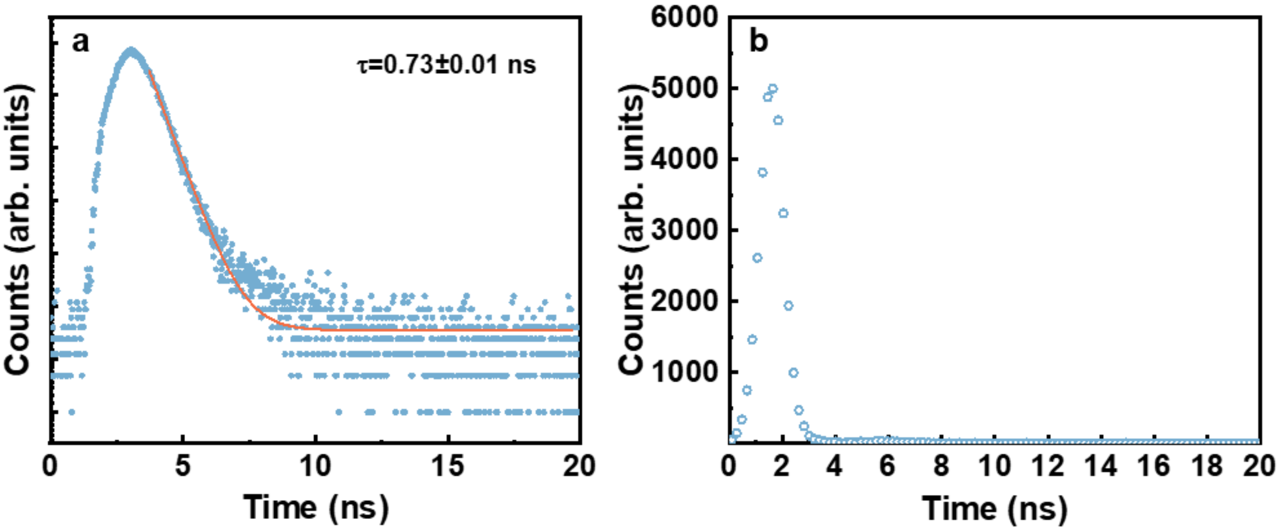

Supplementary Figure 19. PL decay of IR783 at 804 nm under picosecond pulsed laser excitation at 785 nm (a). An estimated singlet lifetime of 0.73 ± 0.01 ns was acquired by fitting the decay process (red solid line). The IRF of the 785 nm pulsed laser was given (b).

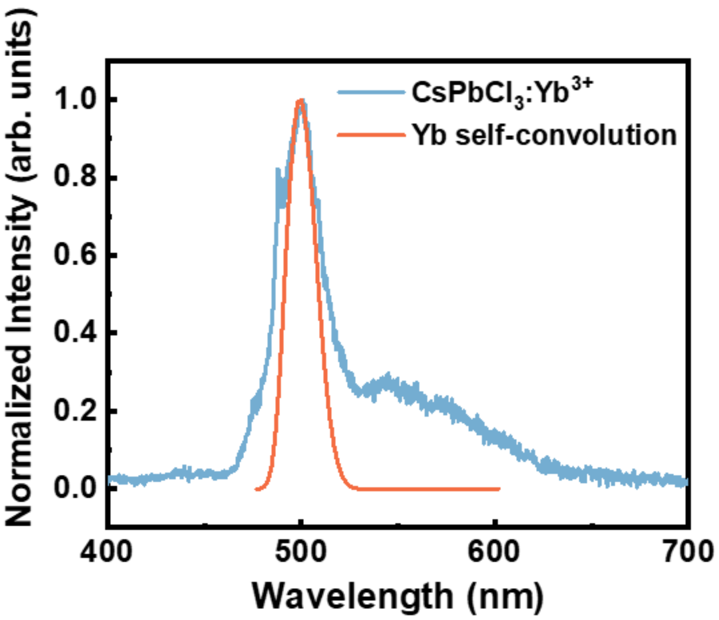

Supplementary Figure 20. Upconversion luminescence from $CsPbCl_3:Yb^{3+}$ nanocrystals under high laser power density (λ_{Ex}=980 nm, power density=2825.3 W/cm^2), in comparison with the self-convolution of Yb^{3+} luminescence.

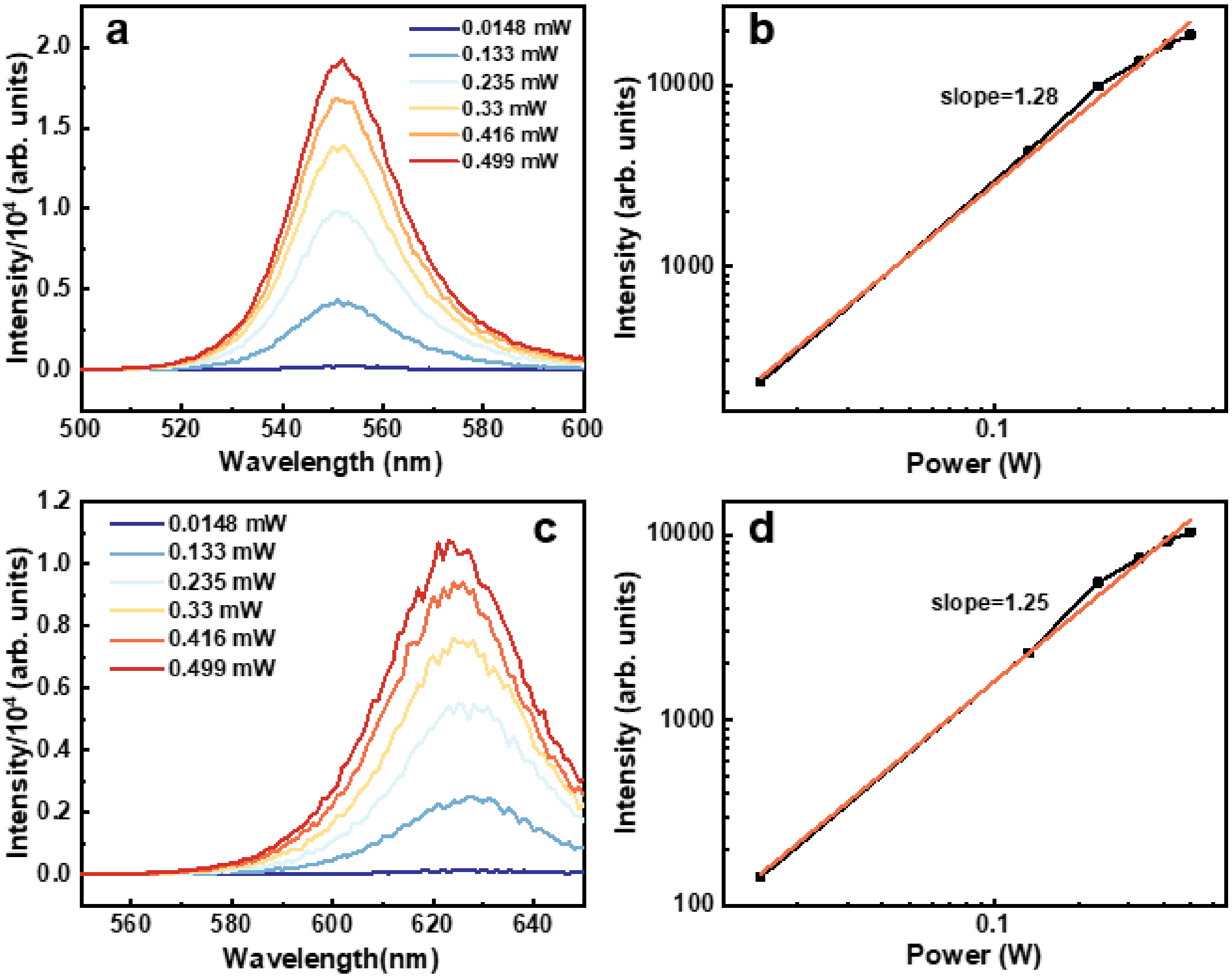

Supplementary Figure 21. Power-dependent upconversion luminescence spectra of IR783-$CsPbBr_2I$ (a) and IR783-$CsPbBrI_2$ (c). The corresponding integrated upconversion luminescence for IR783- IR783-$CsPbBr_2I$ (b) and IR783-$CsPbBrI_2$ (d) as a function of pump laser power is shown on a logarithmic scale. The measured slope values all exceed 1, indicating the involvement of two-photon processes in generating the exciton upconversion luminescence.

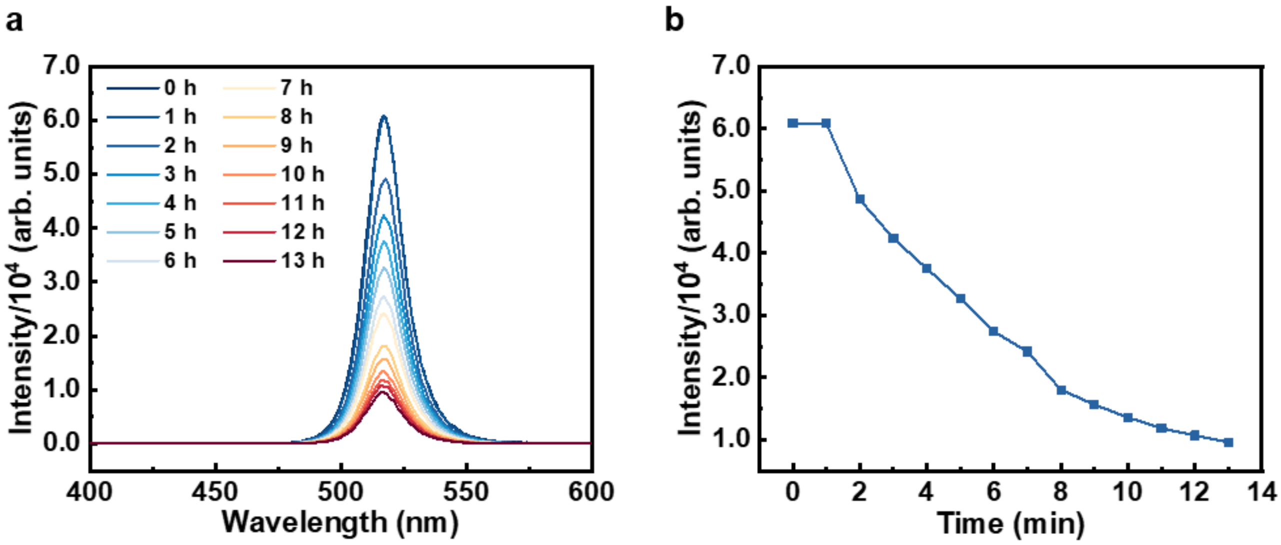

Supplementary Figure 22. The upconversion intensity (a) and its tendency (b) of IR783-$CsPbBr_3:Yb^{3+}$ under daylight radiation (excited by 804 nm laser, power density= 8.4 W/cm^2).

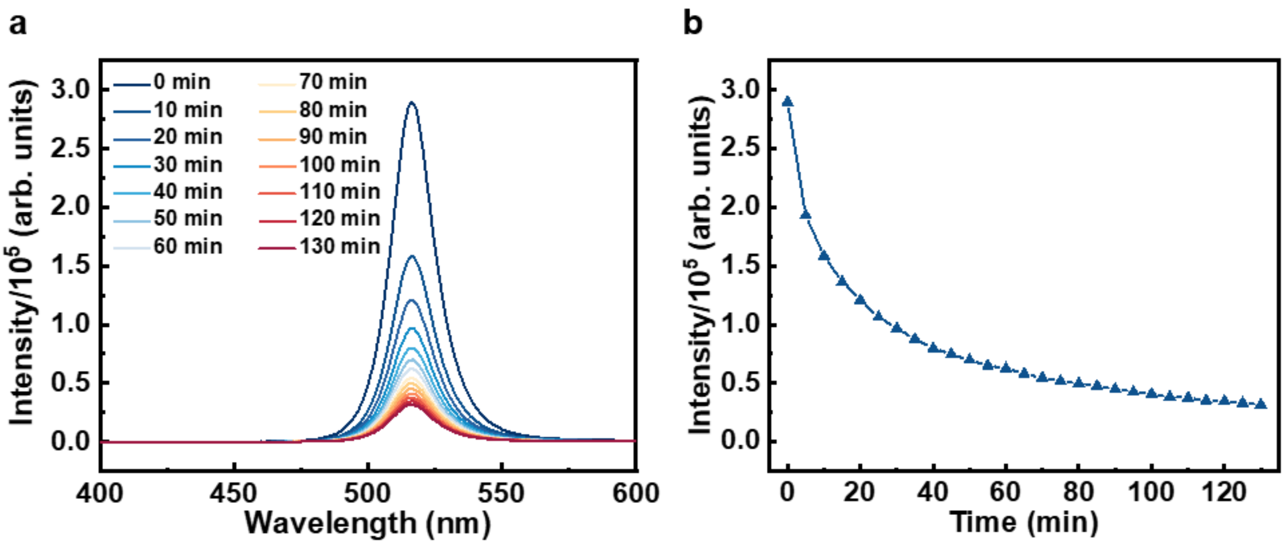

Supplementary Figure 23. The upconversion intensity (a) and its tendency (b) of IR783-$CsPbBr_3:Yb^{3+}$ under 804 nm laser radiation (power density= 8.4 W/cm^2).

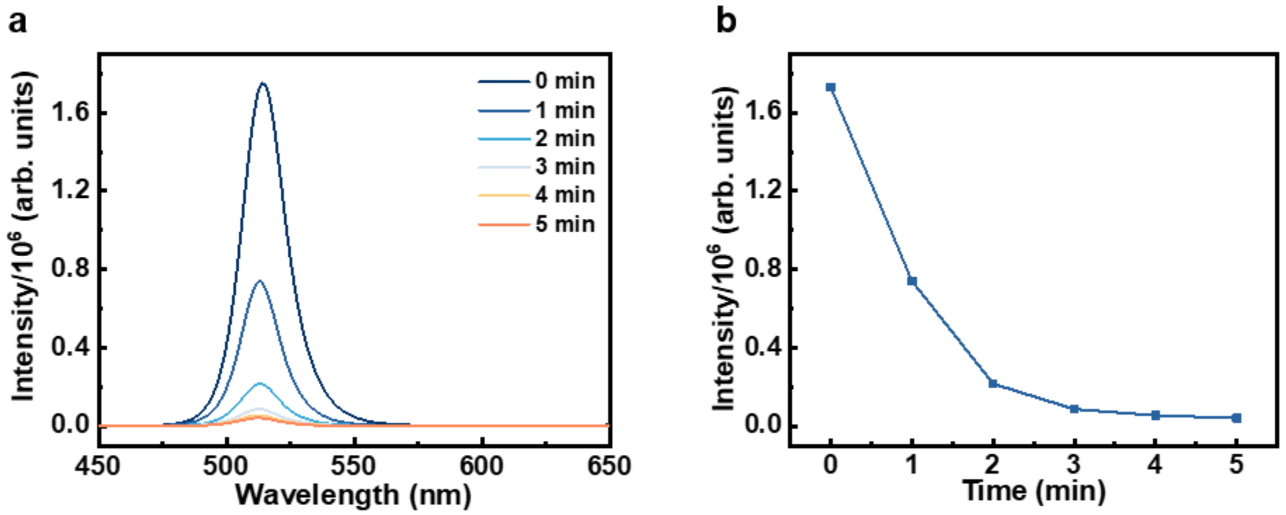

Supplementary Figure 24. The upconversion intensity (a) and its tendency (b) of IR783-$CsPbBr_3$:Yb^{3+} after exposed to ambient atmosphere (excited by 804 nm laser, power density= 8.4 W/cm^2).

Supplementary Tables

Supplementary Table 1. Yb^{3+} doping concentration in PeNCs

sample	Yb/Pb feeding ratio	Nominal Yb doping concentration (mol%)	Actual Yb doping concentration from ICP-OES (mol%)
$CsPbBr_3$	undoped	0.0	0.0
$CsPbBr_3$: $0.08Yb^{3+}$	0.4:1	40	0.80
$CsPbBr_3$: $0.2Yb^{3+}$	1:1	100	1.79
$CsPbBr_3$: $0.3Yb^{3+}$	1.5:1	150	2.91
$CsPbBr_3$: $0.4Yb^{3+}$	2:1	200	3.30

Supplementary Table 2. Comparison of achieved brightness along with their extinction coefficients, quantum yields and the power density with literature-reported well-established dye molecules and upconverting nanocrystals.

	Ex/Em(nm)	ε(M^{-1} cm^{-1})	QY(%)	B (M^{-1} cm^{-1})	PD(W/cm^2)
NCs/IR-783	808/515	261000	0. 0012	3.22	8.4
[1]$LiYbF_4$:0.005Tm^{3+}@$LiYF_4$-$CsPbBr_3$	980/430-580	220000	0.39	858	100
[2]$NaYF_4$:0.2Yb^{3+},0.02Er^{3+}	808/510-570	2880000	0.0001	2.88	533
[3]$NaYF_4$: 0.15Yb^{3+}, 0.2Er^{3+}	970/510-680	4.545	0.11	0.005	181
[2]$NaYF_4$: 0.2Yb^{3+}, 0.02Er^{3+}	980/510-570	1000000	0.0038	38	1128
[4]CH1055	750/1055	10000	0.03	3	downconversion
[5]BTC1070	1014/1070	115000	0.016	18.40	downconversion
[6]Er^{3+}[Zn(II)MC_{quinHA}]	380/1530	55000	$9.9*10^{-4}$	0.5445	downconversion
[7]ICG	785/821	270000	2.4	5400	downconversion
[8]$Eu^{3+}$$[Ligand]^{3-}$	340/605-720	0.067	34	0.0228	downconversion
[9]DM-Naph	441/554	1088889	0.009	9800	downconversion
[10]Rho110	495/520	78000	88	68640	downconversion
[10]Rho560	559/587	97000	12	11640	downconversion
[11]Ag_2S	808/1200	460000	0.08	368	downconversion

Supplementary References

1. Zheng, W.; Huang, P.; Gong, Z.; Tu, D.; Xu, J.; Zou, Q.; Li, R.; You, W.; Bunzli, J. G.; Chen, X., Near-infrared-triggered photon upconversion tuning in all-inorganic cesium lead halide perovskite quantum dots. *Nat Commun* **2018,** *9* (1), 3462.
2. Martins, J. C.; Bastos, A. R. N.; Ferreira, R. A. S.; Wang, X.; Chen, G.; Carlos, L. D., Primary Luminescent Nanothermometers for Temperature Measurements Reliability Assessment. *Advanced Photonics Research* **2021,** *2* (5).
3. Stanton, I. N.; Ayres, J. A.; Stecher, J. T.; Fischer, M. C.; Scharpf, D.; Scheuch, J. D.; Therien, M. J., Power-Dependent Radiant Flux and Absolute Quantum Yields of Upconversion Nanocrystals under Continuous and Pulsed Excitation. *The Journal of Physical Chemistry C* **2017,** *122* (1), 252-259.
4. Antaris, A. L.; Chen, H.; Cheng, K.; Sun, Y.; Hong, G.; Qu, C.; Diao, S.; Deng, Z.; Hu, X.; Zhang, B.; Zhang, X.; Yaghi, O. K.; Alamparambil, Z. R.; Hong, X.; Cheng, Z.; Dai, H., A small-molecule dye for NIR-II imaging. *Nature Materials* **2015,** *15* (2), 235-242.
5. Wang, S.; Fan, Y.; Li, D.; Sun, C.; Lei, Z.; Lu, L.; Wang, T.; Zhang, F., Anti-quenching NIR-II molecular fluorophores for in vivo high-contrast imaging and pH sensing. *Nature Communications* **2019,** *10* (1).
6. Trivedi, E. R.; Eliseeva, S. V.; Jankolovits, J.; Olmstead, M. M.; Petoud, S.; Pecoraro, V. L., Highly Emitting Near-Infrared Lanthanide “Encapsulated Sandwich” Metallacrown Complexes with Excitation Shifted Toward Lower Energy. *Journal of the American Chemical Society* **2014,** *136* (4), 1526-1534.
7. Zhang, K.; Li, H. Y.; Lang, J. Y.; Li, X. T.; Yue, W. W.; Yin, Y. F.; Du, D.; Fang, Y.; Wu, H.; Zhao, Y. X.; Xu, C., Quantum Yield‐Engineered Biocompatible Probes Illuminate Lung Tumor Based on Viscosity Confinement‐Mediated Antiaggregation. *Advanced Functional Materials* **2019,** *29* (44).
8. Starck, M.; Pal, R.; Parker, D., Structural Control of Cell Permeability with Highly Emissive Europium(III) Complexes Permits Different Microscopy Applications. *Chemistry ‐ A European Journal* **2015,** *22* (2), 570-580.

9. Ye, Z.; Yang, W.; Wang, C.; Zheng, Y.; Chi, W.; Liu, X.; Huang, Z.; Li, X.; Xiao, Y., Quaternary Piperazine-Substituted Rhodamines with Enhanced Brightness for Super-Resolution Imaging. *Journal of the American Chemical Society* **2019,** *141* (37), 14491-14495.

10. Zhou, W.; Fang, X.; Qiao, Q.; Jiang, W.; Zhang, Y.; Xu, Z., Quantitative assessment of rhodamine spectra. *Chinese Chemical Letters* **2021,** *32* (2), 943-946.

11. Shen, Y.; Lifante, J.; Zabala-Gutierrez, I.; de la Fuente-Fernandez, M.; Granado, M.; Fernandez, N.; Rubio-Retama, J.; Jaque, D.; Marin, R.; Ximendes, E.; Benayas, A., Reliable and Remote Monitoring of Absolute Temperature during Liver Inflammation via Luminescence-Lifetime-Based Nanothermometry. *Adv Mater* **2022,** *34* (7), e2107764.
